# Clofazimine-Pigmented Intestine

**DOI:** 10.4269/ajtmh.25-0182

**Published:** 2025-07-01

**Authors:** Hideharu Hagiya

**Affiliations:** Department of Infectious Diseases, Okayama University Hospital, Okayama, Japan

**Figure 1.**
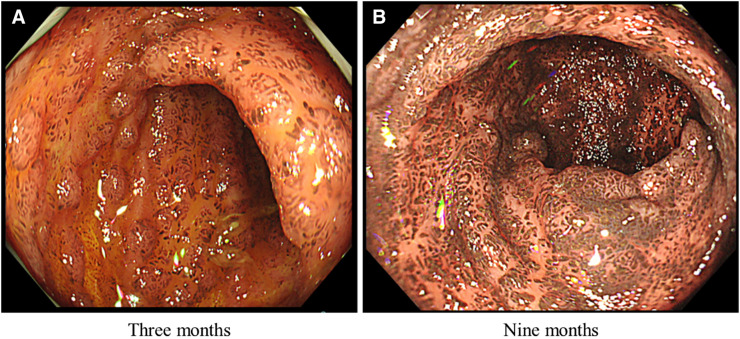
Colonoscopy findings revealing membranous pigmentation in the terminal ileum. (**A**) Three months after clofazimine treatment. (**B**) Nine months after clofazimine treatment.

A 19-year-old Japanese male patient who had been suffering from a disseminated *Mycobacterium intracellulare* infection for several years was referred to our institution. An in-depth genomic investigation of the human DNA sequence confirmed that the patient had primary immunodeficiency attributable to signal transducer and activator of transcription 3 gain-of-function syndrome as the underlying pathological condition. The pathogen demonstrated resistance to primary therapeutic agents, including clarithromycin and azithromycin, necessitating the initiation of combination therapy comprising amikacin, rifabutin, and clofazimine (100 mg/day). Three months later, the patient underwent a lower gastrointestinal endoscopy for malignancy screening, which revealed pigmented intestinal mucosa in the terminal ileum (Figure 1A) and no pigmentation in the colonic mucosa. A follow-up examination 9 months after the initiation of treatment revealed further pigmentation of the mucosa (Figure 1B), despite the absence of any abdominal symptoms. A histopathological examination revealed a deposition of black-purple crystals in the lamina propria. Throughout the treatment course, the patient also developed progressive cutaneous hyperpigmentation, without ichthyosis. Based on these clinical presentations, the terminal ileal pigmentation was considered to be induced by clofazimine administration.

Clofazimine is a riminophenazine compound with antimycobacterial activity against nontuberculous mycobacterial infections, including drug-resistant tuberculosis and leprosy. The drug accumulates in adipose tissues, the reticuloendothelial system, and macrophage-rich organs because of its high lipophilicity and clinically develops skin pigmentation and enteropathy.^1^ Clofazimine-induced enteropathy is classified into three patterns: eosinophilic/allergic enteropathy, a Crohn’s disease-like pattern with granuloma, and crystal-storing histiocytosis.^2^ These adverse effects possibly develop when clofazimine is administered at dosages exceeding 100 mg daily, resulting in abdominal pain, diarrhea, and gastrointestinal hemorrhage. Differential diagnosis of intestinal pigmentation is limited; however, melanosis ilei, which occurs in patients receiving oral iron supplementation or activated charcoal administration, warrants consideration.^3^^,^^4^ Clinicians should maintain awareness of this rare adverse manifestation when prescribing clofazimine.^5^
